# Supplementary material for: Male Pheromones Induce Ovulation in Female Honeycomb Groupers (Epinephelus merra): A Comprehensive Study of Spawning Aggregation Behavior and Ovarian Development
Source: Cells. 2022 Jan 30;11(3):484. doi: 10.3390/cells11030484 (PMC8833890; doi:10.3390/cells11030484)
Supplement: Supplementary file 1 [file cells-11-00484-s001.zip › cells-1453773-supplementary.pdf]

Supplementary Table S1. Sequences of primers for quantitative real-time PCR.

Gene		Sequence(5' - 3')	Amplicon
fsh-β	Forward	CTGTGGCCAGAAACTGCAAG	120
	Reverse	GTCAATGTAGGACGGACGGG	
	Probe	GCAGGAAACACGTACTGCGGGCG	
fsh-β (standard)	Forward	GGGCTGAACAGAAAGTCTGC	228
	Reverse	TCAAGTCATGTGACACAAAGATAA	
lh-β	Forward	GTGATGTTCCCTTTGATGTTGAGT	
	Reverse	CACTTTGGACAGCCTTCCTTCT	137
	Probe	TGCAGCGGCCTTCCAGCTGC	
lh-β(standard)	Forward	TACAGGTCGGCAGAGTGATG	218
	Reverse	CTTGATGACAGGGTCCTTCG	

Supplementary Table S2. Egg collection in rearing tanks and oocyte stages in females.

Experimental group	n	24h	48h	72h
Control	7	-<br>NI	-<br>NI	-<br>TY-I (6), AO(1)
MW	7	-<br>NI	-<br>NI	+<br>TY-I (4), OV(3)

-, no eggs in the rearing tank: +, eggs were collected. TY-I, tertiary yolk stage-I: OV, ovulatory egg stage: AO, atretic oocyte stage. ( ) shows the number of individuals in each oocyte stage. NI, no individual sampling.
